# Qualitative analysis of the coding of pathological data of cancer registry centers: A study in North of Iran

**DOI:** 10.1371/journal.pone.0291139

**Published:** 2023-09-15

**Authors:** Mohammad-Ali Jahani, Ghahraman Mahmoudi, Hossein-Ali Nikbakht, Zeinab Farhadi, Raziehsadat Mousavi

**Affiliations:** 1 Health Research Institute, Social Determinants of Health Research Center, Babol University of Medical Sciences, Babol, Iran; 2 Hospital Administration Research Center, Sari Branch, Islamic Azad University, Sari, Iran; 3 Department of Health Services Management, PhD Student in Health Services Management, School of Health Management and Information Sciences, Iran University of Medical Sciences, Tehran, Iran; Brazilian national cancer institute, BRAZIL

## Abstract

**Background:**

The cancer registry system is an important part of the cancer control program. Improper coding of cancers leads to misclassification and incorrect statistical information about cancer. Therefore, in this study, the main objective of the qualitative analysis was the accuracy in assigning the codes to the pathological reports in the centers responsible for cancer registry.

**Methods:**

This study was descriptive, retrospective and applied. The data source in this study included 15,659 pathology reports received during the years 2017–2019 in the population-based cancer registry centers of Mazandaran province. Out of 1800 reports, 1765 samples of reports were selected and analysis was done on them by stratified random sampling method. A researcher-made checklist was used to collect data, and the Kappa agreement coefficient and Cohen’s agreement percentage were presented to check the accuracy of the reports. STATA13 was used for data analysis.

**Results:**

1150 of 1765 pathology reports (65.0%), did not have a topographic, morphological and behavioral codes and 410 (23.2%) had grade codes. The Kappa coefficient in reports with a topography code was 0.916 and with a morphology code it was 0.929, respectively. In behavior coding, the highest agreement is in the category of benign cancers at 65.2% and in grade coding in the category without grade is 100%.

**Conclusion:**

The most reports were on carcinoma morphology, and the Kappa coefficient in morphology codes has almost complete reliability. In terms of behavior coding, there was the most agreement in the category of benign cancers. The Kappa coefficient in given behavior codes has low reliability.

## Introduction

In the past few years, significant changes have been seen in the occurrence of diseases. One of the most important changes is the reduction of the burden of diseases that are transmitted in a contagious way and the increase in, prevalence, incidence and mortality of non-communicable diseases [1]. Cancer is an important non-communicable diseases and one of the main causes of death in the world [2]. To reduce the prevalence and occurrence of cancer, programs are needed to control this disease. One of the most important things that helps control it is cancer registry [3]. Data related to cancer incidence and mortality due to it is widely used for research on the cause of cancer, control and planning in health care [4]. Published reports from developed and developing countries provide up-to-date information on cancer incidence, trends and prognosis [5]. Despite the significant disease burden and increasing risk factors, the need for effective use of limited resources is felt and, as a result, the establishment of a national cancer control program is always recommended [6]. Today, cancers are one of the most important priorities in the health sector. Iran is considered and attention to these types of diseases and planning to control them is very necessary and important [7].

The first step in cancer control is to collect accurate and complete information and statistics from cancer patients, which can be achieved in the form of a population-based cancer registry program [8]. Gathering accurate and complete information from cancer patients is in fact the first and is the most important step in designing a comprehensive cancer control program in Iran [9]. Due to the correct classification of cancer data, it has an important effect in determining cancer patterns in the population of each region, disease and treatment trends, planning and evaluating cancer control programs, prioritizing the allocation of resources for cancer, and conducting clinical research and cancer epidemiology [10,11]. In the ICD-O-3 classification by the World Health Organization, it provides standards for coding topography, tumor location, morphology, microscopic shape of the tumor, malignant tumor behavior, in origin or benign, degree and degree of tumor differentiation [12]. Making any mistake in coding causes a mistake in the classification of the disease and causes errors in its statistics [13,14]. Coding is the assignment of code symbols on a contract basis instead of the concepts contained in diagnoses and expressions [15], topographic code indicating the position of the source of cancer [16], morphological code, code assigned to the type of cancer of the affected cell [17] and its behavior indicates the invasive or non-invasive nature of the neoplasm [18] and the grade code indicates how similar the tumor is to normal tissue [19].

Due to the importance of the subject, studies were conducted in this field: in the study of Beam et al., the correctness of the codes according to the international classification system of diseases in the diagnosis of bronchopulmonary dysplasia in the electronic health record did not exist. It is used to conduct research and also to provide health services on a large scale [20]. In a study by Guo et al., It was stated that documenting disease classification codes promotes electronic health records [21]. In Jansen’s study, based on the data obtained from the cancer registry, it is proposed to try to improve the quality indicators related to the diagnosis of diseases, the necessary follow-ups and the treatment of cancer [22]. In the study by Lyu et al., Only 59.8% of sarcoma patients were accurately coded according to the ICD-O-3 classification, and 2.5% were coded with another cancer diagnosis [23]. In the study by Pukkala et al., cancer registry data in Northern European countries, Finland, Denmark, Norway Iceland and Sweden had a high standard of accuracy and completeness [24].

Therefore, considering the increasing incidence of cancer and the importance of early diagnosis of the disease in the early stages, the quality and accuracy of coding information of cancer patients is of great importance. For this reason, in this research, the coding analysis of the pathological data of cancer registry centers based on the population of Mazandaran Universities of Medical Sciences has been done. It is expected that the quality of any data in the cancer registry can be improved by measuring the quality of assigned codes as an important reference in the national cancer control program and identifying possible weaknesses and providing solutions to improve registry management processes and finally lead to more complete and accurate statistics and determination of disease burden in the field of common and priority cancers, as well as better planning and decision-making by managers in the region.

## Methods

This study was descriptive, retrospective and applied. It was done with a letter of introduction and code of ethics under the number IR.IAU.CHALUS.REC.1399.025, which was obtained from Islamic Azad University. In accordance with the approval of the Islamic Council of the Islamic Republic of Iran, the reports of cancer patients are registered in the cancer registry centers of the universities of medical sciences in the country, and this information is available for researchers in the field of cancer, by completing legal procedures and obtaining a code of ethics and permission to use data. In this research, through the mentioned steps, the pathology information of cancer patients was provided to the researcher without mentioning the first names and surnames of the patients, and for this reason, there was no need to obtain the consent of the participants in the study.

The data source in this study included 15,659 pathology reports that were sent to cancer registry centers based on the population of Mazandaran Universities of Medical Sciences during the years 2017–2019, which was sent to this center by all public and private pathology centers under the auspices of the university and through the cancer registry system. Due to the fact that collecting, repeating, coding and analyzing reports of cancer patients from relevant centers is a time-consuming process, according to the international standard in the cancer registry program, it always takes 3 to 5 years to complete this process. Therefore, all the research done on these reports is always related to the reports of 3 years ago. In this study, the evaluation was done only on the pathology reports. It means the pathology reports were coded in the pathology centers, and then they were studied and coded again in the cancer registry center and finally the comparison was done between two groups of codes.

Using Cochran’s formula to estimate the ratio in the community in descriptive-analytical studies and using a study that reported a rate of coding error between pathology centers and the first coder (0.22) [25]. Considering the reliability level of 0.95 and the power of the study of 0.90 and the acceptable error of 0.035 for the ratio, at least 1500 samples were required. For this reason, approximately 11% of samples from each year (of the three years studied) were considered. Finally, 1800 pathology reports were analyzed in this study. In total, 12 reports were excluded from the study due to the fact that they were from pathology centers outside the region covered by the population of this study, and 23 cases were excluded from the study due to the benign nature of the cancer, and finally, 1765 cases were evaluated and analyzed.

The research method in this research is stratified random sampling. In this way, we had 19 government centers and 60 private centers, and the sample size was sampled in each year, based on the weight of the type of center in the research community. In this way, the number of sampling in three equal clusters for the three years of the study was considered, and in each year, the weight of public and private centers was proportional to the weight of public and private centers in society. Also, the checklist prepared by the researcher to collect data and measure the validity of the checklist from experts and experts were used and approved after making the necessary corrections. Experts mean people who have at least 6 months of experience as cancer registrars. For each record, a code was determined so that the patient’s identity information would not be revealed, and the principle of patient information confidentiality was fully respected.

The researcher-made checklist had three sections. The first part includes: report number, year of report, type of governmental or private center, city of the place of reporting and the initial type or metastasis of the tumor, and the second part includes: information about gender and work history, age, degree and field of study of the cancer registrars. The information related to the second part was obtained by contacting the cancer registrars in the cancer registry pathology center and entered in the checklist. The third part contained information about the coding and evaluations of the correctness and reliability of the codes. Then, according to the location, behavior, grade and histology of the cancer cell present in the patient and referring to the cancer ICD-O-3 coding book, the appropriate code was selected and recorded in the relevant checklist.

### Data analysis and method

To describe quantitative data, central (such as mean) and dispersion (such as standard deviation) indicators were used, and to describe qualitative data, percentage and frequency were used. Also, appropriate tables and graphs were used to better display the data of this research. Cohen’s kappa was used to calculate the reliability of cancer diagnosis codes, and the grading provided by Landis and Koch was used to determine the reliability status using Cohen’s kappa coefficient [26]. In other words, this index indicates the degree of agreement between two evaluators (excluding cases of chance) on a two-mode feature. The kappa coefficient and statistical analysis based on it are numerical values between -1 and +1. The closer it is to +1, it indicates the existence of greater and proportional agreement, and the closer it is to -1, it indicates indirect and inverse agreement. Also, sizes close to zero indicate a lack of agreement, and when the Kappa coefficient is below zero, the degree of reliability is weak, between (0.00–0.20) low, (0.40–2.40) relatively weak, (0.41–0.60) medium, (0.61–0.80) is acceptable and (0.81–1.00) is acceptable.) is almost complete.


Kappa=Pi=(PA0–PAE)/(1–PAE)

The amount of agreement between two evaluators is determined by the value of PA0, and the value of PAE indicates the expected agreement [15]. The percentage of agreement was shown by calculating the kappa coefficient. Logistic regression model was also used to investigate the relationship between demographic characteristics of people in pathology centers with correct and incorrect registry of topographical codes. Also, the odds ratio (OR) and 95% confidence interval (CI) were used to show the effect size in the model and the significance level (P<0.05) was considered. STATA software version 13 was used to analyze this study.

## Results

In this study, 1765 pathology reports were reviewed (0.65%), of which 1150 cases had no topographical, morphological and behavioral codes and only 410 cases (23.2%) had grade codes. These reports were obtained from 13 cities that had active pathology centers. The mean and standard deviation of the age of cancer registrants of pathology reports was 38.66 ± 7.59 years.

Most of the cancer registrars were 1388(78.6%) women, 1113 (63.1%) of the cancer registrars had a degree in laboratory sciences and the highest level of education with 892 (50.5%) bachelor’s degree, also, 964 (54.6%) of the registered reports were related to governmental centers. Complete demographic characteristics of pathology cancer registrars include; gender, age, work experience, field and degree were presented in (Table 1).

**Table 1:** The level of accuracy of the topography code according to the demographic characteristics of the pathology centers.

Variable Type	Variable Subgroup	Frequency (percentage)	Frequency (percentage)	The accuracy of the topographic code		
				Kappa coefficient 1765 = Number	Kappa coefficient 615 = Number	Percentage of agreement 615 = Number
**Gender**	**Male**	377 (21.4)	165 (26.8)	0.216	0.500	52.73
	**Female**	1388 (78.6)	450 (73.2)	0.204	0.644	65.78
**Age Category**	**Less than 50**	1675 (94.9)	555 (90.2)	0.204	0.629	64.50
	**50 and more**	90 (5.1)	60 (9.8)	0.261	0.394	41.67
**Work Experience**	**Less than 15**	878 (49.7)	359 (58.4)	0.246	0.615	63.23
	**15 and more**	887 (50.3)	256 (41.6)	0.168	0.594	60.94
**Major**	**Laboratory sciences**	1113 (63.1)	361 (58.7)	0.182	0.572	59.00
	**Microbiology**	190 (10.3)	16 (2.6)	0.070	0.845	87.50
	**Pathology**	71 (4.0)	50 (8.1)	0.292	0.415	44.00
	**Biology**	137 (7.8)	46 (7.5)	0.137	0.405	43.48
	**Other medical sciences**	8 (0.5)	0 (0.0)	-	-	-
	**Other non-medical sciences**	246 (13.9)	142 (23.1)	0.450	0.794	80.28
**Grade of Study**	**Diploma**	150 (8.5)	110 (17.9)	0.558	0.770	78.18
	**Associate Degree**	391 (22.2)	93 (15.1)	0.100	0.423	45.16
	**Bachelor’s Degree**	892 (50.5)	279 (45.4)	0.177	0.579	59.50
	**Master’s Degree**	261 (14.8)	83 (13.5)	0.242	0.783	80.72
	**Specialist**	71 (4.0)	50 (8.1)	0.292	0.415	40.00
**Type of Center**	**Governmental**	964 (54.6)	346 (56.3)	0.224	0.636	65.03
	**Private**	801 (45.4)	269 (43.7)	0.186	0.568	58.74
**Total**		1765 (100)	615 (100)	0.207	0.607	62.28

According to the announcement of the exact codes made by the coder of the Cancer Registry Center, the total kappa coefficient in these cases was 0.607 (average accuracy) and the total agreement in these codes was 62.28%. From 615 declared codes, the kappa coefficient of reports in government centers was 0.636 (acceptable accuracy) and the percentage of agreement was 65.03% and the kappa coefficient of private centers was 0.568 (average accuracy) and the agreement was 58.74%. The accuracy of the topographic code in terms of demographic characteristics was presented in Table 2.

**Table 2:** Assessing the accuracy of the topographical codes given by pathology centers by site of cancer.

ROW	Classification ICD-O	Topographic category	Total number of the reports received = 1765			Number of reports with topographic codes = 615		
			Cancer Registration Expert Coding (percentage) frequency	Coding of pathology cancer registrars (percentage) frequency	Coding of pathology cancer registrars (percentage) frequency	Cancer Registration Expert Coding (percentage) frequency	Coding of pathology cancer registrars (percentage) frequency	Internal reliability Percentage agreement (percentage) frequency
**1**	**C00-C14**	**Lips, oral cavity and throat**	30 (1.7)	8 (0.4)	8 (26.7)	12 (1.4)	14 (1.6)	12 (100)
**2**	**C15-C26**	**Digestive organs**	576 (32.6)	253 (14.3)	246 (42.7)	356 (1.2)	356 (41.2)	345 (98.0)
**3**	**C30-C39**	**Respiratory system and organs inside the chest**	36 (2.0)	4 (2.0)	4 (11.1)	10 (1.2)	9 (1.1)	9 (90.0)
**4**	**C40-C41**	**Bones, joints and articular cartilage**	14 (0.8)	6 (0.3)	6 (42.9)	10 (1.2)	7 (0.8)	7 (70.0)
**5**	**C42**	**Blood systems and hematopoietic organs**	84 (4.8)	2 (0.1)	2 (2.4)	7 (0.8)	7 (0.8)	7 (100)
**6**	**C44**	**Skin**	161 (9.1)	57 (3.2)	54 (33.5)	78 (9.0)	80 (9.2)	76 (98.7)
**7**	**C49**	**Soft and connective tissue**	10 (0.6)	3 (0.2)	3 (30.0)	3 (0.4)	3 (0.4)	3 (100)
**8**	**C50**	**Breast**	219 (12.4)	88 (0.55)	84 (38.4)	125 (14.4)	118 (13.6)	112 (91.1)
**9**	**C51-C58**	**Female genitals**	135 (7.6)	79 (4.5)	72 (53.3)	88 (10.2)	94 (10.9)	83 (95.4)
**10**	**C60-C63**	**Male genitals**	122 (6.9)	39 (2.2)	38 (31.1)	62 (7.1)	58 (6.7)	45 (88.5)
**11**	**C64-C68**	**Urinary system**	170 (9.6)	36 (2.0)	32 (18.8)	52 (6.0)	53 (6.1)	48 (94.1)
**12**	**C69-C72**	**Eyes, brain and other parts of the central nervous system**	38 (2.2)	2 (0.1)	2 (5.3)	2 (0.2)	2 (0.2)	2 (100)
**13**	**C73-C75**	**Thyroid and other endocrine glands**	23 (1.3)	2 (0.1)	2 (8.7)	8 (0.9)	8 (0.9)	8 (100)
**14**	**C76**	**Ill- defined positions**	4 (0.2)	4 (0.2)	2 (50.0)	2 (0.2)	5 (0.6)	2 (100)
**15**	**C77**	**Lymph nodes**	49 (2.8)	10 (0.6)	5 (10.2)	10 (1.2)	15 (1.8)	9 (90.0)
**16**	**C80**	**Unknown primary positions**	94 (5.3)	18 (1.0)	8 (8.5)	40 (4.7)	36 (4.2)	22 (55.0)
**Kappa coefficient**			**0.280**			**0.916**		
**Percentage of total agreement**			**39.43**			**93.45**		

Based on the standard classification of cancers, the Kappa coefficient was 0.280 (relatively good) in all reports and 0.916 (almost perfect) in the reports received with topography code, respectively. Also, the percentage of total agreement for these two cases was 39.43 and 93.45%, respectively. In addition, the rate of agreement was presented separately in the group of cancers in (Table 2).

According to the standard classification of cancers, among the 1765 reports of morphological codes coded by the cancer registry expert of the University Cancer Registry and its measurement with the morphological codes coded by the pathology cancer registrars, the most reports on cancer morphology, percent of the agreement in morphological codes was 99.3%.Also, the kappa coefficient in the given morphological codes was 0.929 (almost complete reliability) and the percentage of total agreement was 99.18%. In terms of coding of behavior, the most agreement was in the category of benign cancers and at the rate of 65.2%. The kappa coefficient in the registered codes, given between the expert cancer registrar of the university cancer registry and pathology cancer registrars, was 0.020 (low reliability) and the percentage of total agreement was 35.35. In terms of grade coding, there was the most agreement in the category of grade 0 (no grade) and 100%, kappa coefficient in the codes that were coded by the expert cancer registrar of the university cancer registry and pathology cancer registrars was 0.067 (low reliability) and the percentage of total agreement was 19.19 (Table 3).

**Table 3:** Accuracy of morphology, behavior and grades codes given by pathology centers.

Row	Classification ICD-O	Cancer Registration Expert Coding (percentage) frequency	Coding of pathology cancer registrars (percentage) frequency	Internal reliability Percentage agreement (percentage) frequency	Kappa coefficient	Percentage of total agreement
**Morphology codes**	**Carcinoma**	570 (93.8)	573 (94.2)	569 (99.3)	0.929	99.18
	**lymphoma**	5 (0.8)	5 (0.8)	5 (100)		
	**leukemia**	0 (0.0)	0 (0.0)	-		
	**Other specific cancers**	16 (2.6)	16 (2.6)	16 (100)		
	**Unspecified cancers**	17 (2.8)	14 (2.3)	13 (92.2)		
**Behavior codes**	**Benign**	27 (1.5)	1150 (65.2)	27 (65.2)	0.020	35.35
	**Unknown**	14 (0.8)	2 (0.1)	1 (0.7)		
	**In situ**	24 (1.14)	8 (0.5)	0 (0.0)		
	**Malignant**	1700 (96.3)	605 (34.3)	596 (35.1)		
**Grade codes**	**Grade 0 (no grade)**	30 (1.7)	1355 (76.8)	30 (100)	0.067	19.09
	**Grade 1**	136 (7.7)	35 (2.0)	17 (12.5)		
	**Grade 2**	260 (14.7)	32 (1.18)	20 (7.7)		
	**Grade3**	181 (10.3)	39 (2.2)	22 (12.2)		
	**Grade 9 (Unknown)**	1158 (65.6)	304 (17.2)	248 (21.4)		

According to the regression model conducted in the study of the relationship between the demographic characteristics and the error rate in the registry of topographical codes, it showed that the most errors in the registry were related to the demographic characteristics of the cancer registrants. The results showed that the rate of error in registering topographic codes of private centers is higher so that the chance of error in registering reports of topographic codes of private centers was 34% higher than governmental centers. Although this relationship was not statistically significant, it was close to a significant level. Also, women are more accurate and have a lower error rate in registry, so that the chance of having a registry error in women is 44% lower than men, and this relationship was statistically significant. The average error in registering codes in people over 50 and 50 years old is more than 2.5 times that of people under 50 years old. Also, people with a diploma had the lowest error rate in registry, on average, the chance of error in registry in people with associate, bachelor and master and higher degree was more than 4.6, 1.5 and 1.9 times higher than people with a diploma (Table 4).

**Table 4:** The results of the regression model in examining the relationship between demographic characteristics and the error rate in recording topographical codes between cancer registrars of the university and pathology centers.

Variable	Variable subgroups	Frequency (percentage)	The result of the review		(95%CI) OR	P-value
			Accurate Frequency (percentage)	Error Frequency (percentage)		
**Type of center**	**Governmental**	346 (56.3)	227 (65.6)	119 (34.4)	Reference group	0.081
	**Private**	269 (43.7)	158 (58.7)	111 (41.3)	(1.18–0.96) 1.34	
**Gender**	**Man**	165 (26.8)	87 (52.7)	78 (47.3)	Reference group	0.002
	**Female**	450 (73.2)	298 (66.2)	152 (33.8)	(0.81–0.39) 0.56	
**work experience**	**Less than 15 years**	359 (58.4)	229 (63.8)	130 (36.2)	Reference group	0.471
	**15 and more**	256 (41.6)	156 (60.9)	100 (39.1)	(1.57–0.81) 1.12	
**Age**	**Less than 50 years**	555 (90.2)	360 (64.9)	195 (35.1)	Reference group	0.001
	**50 and more**	60 (9.8)	25 (41.7)	35 (58.3)	(4.44–1.50) 2.58	
**Major**	**Laboratory sciences**	361 (58.7)	215 (59.6)	146 (40.4)	Reference group	0.063
	**Other**	254 (41.3)	170 (66.9)	84 (33.1)	(1.01–0.52) 0.72	
**Grade**	**Diploma**	110 (17.9)	87 (79.1)	23 (20.09)	Reference group	
	**Associate Degree**	93 (15.1)	42 (45.2)	51 (54.8)	(8.49–2.48) 4.59	0.001>
	**Bachelor’s degree**	279 (45.4)	167 (59.9)	(40.1)112	(4.25–1.51) 2.53	0.001>
	**Master’s degree and higher**	133 (21.6)	89 (66.9)	44 (33.1)	(3.35–1.04) 1.87	0.036

## Discussion and conclusion

In this study, the results show that from the reports that were received from the pathology centers and had codes, those coded by cancer pathology registrars with female gender, age group less than 50 years, work experience less than 15 years, field Microbiology, M.Sc., and M.Sc., who were coded in government centers, had higher accuracy and precision. In the reports that had a topography code, the highest percentage of cancers were related to gastrointestinal cancers, breast cancer, female genital tract cancer, and skin cancer. In reports coded for morphology, code accuracy on coded reports was nearly perfect, but code accuracy for behavior and grade was low, even in declared codes.

The results of this study showed that based on the registry of accurate topographical codes of 615 announced, the kappa coefficient of reports in public centers is 0.636 (acceptable accuracy) and the agreement percentage is 65.03% and the kappa coefficient of private centers is 0.568 (average accuracy) and the agreement rate was 58.74%.

The results of Woodfield et al.’s study showed that although PPV (Positive Predictive Value)s for stroke and its pathological types ranged from 6–97%, appropriately selected, stroke-specific codes (rather than broad cerebrovascular codes) consistently produced PPVs >70%, and in several studies >90% [27].Beam stated in a study about the international classification of diseases in bronchial dysplasia that the accuracy of each code is from 82 to 95% [20].

The Kappa coefficient in all reports and reports received with a topography code was 0.280, according to the standard classification of cancers (relatively good) and 0.916 (almost complete). Also, the percentage of total agreement for these two cases was 39.43 and 93.45%, respectively.

Maryati’s study shows that the quality of coding of medical records is desirable, which, of course, can be improved by improving the quality of medical records [28]. In the study of Rachmad about the Accuracy of Internal Medicine Clinical Patient Diagnostic Coding, some errors in coding are caused by unclear writing of disease diagnoses, incomplete writing of disease diagnoses, inaccuracy in determining the main diagnosis, and lack of communication between the medical record officer in the coding section with nurses or doctors [29]. Lyu et al also argued that one of the most important causes of coding errors is the vague definitions of the disease. For this reason, national data may not be sufficiently robust and complete for population-based studies in sarcoma [23]. In Iran, the policy of the cancer department in the Ministry of Health and Medical Education is that cancer registry in pathology centers is done by directly registering cancer codes online in the same place and in this way previous studies show that cancer registry data does not have the desired quality [30,31].

But this study shows the correctness of the codes sent to the reports sent to the cancer registry centers in the north of the country, and the reason for this could be the access to oncology and pathology experts who participate as consultants in the cancer registry program and the necessary meetings and it is continuously formed to remove ambiguity in cancer diagnosis and to assign appropriate and correct codes for them. On the other hand, malignancy reports from pathology centers are registered online on the cancer registry system of Mazandaran Universities of Medical Sciences, and these reports are monitored daily by cancer registry experts, and if there are any problems in the registry, they are immediately reported to the pathology centers. So, the mistakes in this registry will be corrected as soon as possible and this will increase the accuracy of the coding. Also, the presence of many cancers with similar topography can be the cause of low error in their topography coding.

The results showed that a significant percentage of the reports that were considered for analysis in this study (65%) did not provide topographical, morphological or behavioral codes, which indicates the incomplete coding that was done in pathology centers. It is caused by the lack of knowledge of the coders for accurate and complete coding in pathology centers. Also, among 1765 morphological coding reports by the expert cancer registrar of the university cancer registry and its measurement with morphological coding reports by pathology cancer registrars, the percentage of agreement was 99.18%. The percentage of total agreement in the codes registered by the expert cancer registrar of the university cancer registry and pathology cancer registrars was 35.35 and in the grade codes were 19.09. The Sollie study on quality assessment of cancer registries in Dutch primary care shows that 60% of the reports of cancer patients are fully coded in accordance with NCR [32]. In Wanner et al. study aim to evaluate and measure the quality of data in the cancer registry, it is stated that all the cases of malignant cancer (except for non-melanoma skin cancer) are known in the country that were obtained from 1980 to 2014 and the results indicated the occurrence of all tumors in this country, which was accompanied by increasing fluctuations over time [33].

Derry Study on Associations between Anxiety, Poor Prognosis, and Accurate Understanding of Scan Results among Advanced Cancer Patients shows that 68% of the medical documents of cancer patients were accurately recorded and reported [34]. Muslimah’s study declared that coder inaccuracy in coding, officers have not implemented coding procedures in neoplasm cases and in assigning codes to neoplasm cases, officers should code according to the SOP(Standard Operating Procedure), so that the resulting code is complete and accurate [35]. In Turner et al. study stated that the cause of death of men who had prostate cancer is correctly reported on their death certificate, and therefore the data is reliable for use in the cancer registry program [36]. Fatimatullailin’s study stated that there are codes that are not accurate because some codes have been memorized by the coder due to the disease codes that often appear so that they do not open the ICD-10, the coder does not match the main diagnosis with the therapy given, the coder does not know DM complications and other diagnoses that occur in cases of diabetes mellitus [37].

Overall, this study indicates an acceptable quality of data in the cancer registry program. This study states that the behavior of each cancer is in one of the three groups: benign, in situ, and invasive or malignant, and among these three groups, only cancers with aggressive behavior have graded. Therefore, one of the coding errors can be considered the registry of grade for benign and in situ tumors, which was not observed in these codes. Also, the data from the research indicates the correctness of the morphological codes that were sent to the cancer registry centers of the universities of medical sciences, and on the other hand, the reason for the low accuracy of the behavioral codes is the lack of registry of their codes in the pathology centers. Accurate and complete coding of disease is very important and all hospitals must record complete and correct coding of disease diagnosis in the relevant software or system, therefore, correct coding of patients’ files is one of the most important duties of coding personnel. Managers should keep this in mind to ensure the quality of their given codes. Their classification is poor and thus poses a risk to caregivers and managers in policy making and planning, research and education, reimbursement and care delivery. The results of the regression model in examining the relationship between the number of errors in registering topographical codes and demographic characteristics state that there is a possibility of error in registering topographical codes show that the chance of error in recording reports of topographic codes of private centers is 34% higher than government centers, the chance of error in registry in women is 44% less. For men, the average error in registering codes in people over 50 is more than 2.5 times that of people under 50, the chance of error in registry in people with an associate degree, bachelor’s degree and master’s degree is up to 4.6, 1.5 and 1.9 times more than people with a diploma. Treister-Goltzman et al., in their study stated that In planning for human resources, there is a need to have a broad perspective on cooperation between governmental and non-governmental organizations [38].Al-Husban recommends that managers in Jordanian private hospitals should hold training courses for all hospital employees to use information systems and should focus continuously on improving the training level of personnel and maintaining these courses [39]. Beazley stated that in hospitals, the personnel of the health information management department, according to their field of activity, should acquire specialized skills in the relevant field [40].

Oetari’s study showed that Human Resources in Medical Record Installations are still lacking and medical record file processing training has not been carried out thoroughly to officers. Facilities and infrastructure to support the work of officers are inadequate. In the assembling section, there are still incomplete documents. Coding officer had difficulty in reading the doctor’s writing and the diagnosis was not found in the ICD-10 book [41]. In general, one of the reasons that the accuracy of coding in private centers is low is the lack of complete coding in these centers. It is necessary to add medical records officers and infrastructure in the Medical Record Intalation. In most private centers, coders have not received specialized training for this task and did not pass academic studies related to coding, so it is necessary to hold courses. Motivational and educational factors for people working in private pathology centers, especially men, as well as employees over 50 years of age and more than 15 years of work experience, should be given more attention so that these codes can be recorded more accurately and improve the quality of data. Also, if special attention is paid to the inclusion of behavioral codes and grades in the integrated hospital system, it is possible to improve the accurate recording of these codes in the pathology reports and hospital records of patients.

One of the limitations of this study is the lack of complete collection of all malignant pathology reports in the three years studied by the pathology centers due to the lack of correct and complete separation of the malignant reports which is done by the cancer registrar in the pathology center to be sent to the cancer registry center. Another limitation in this study is that a number of pathology centers did not fully cooperate to complete the coding of malignancy reports before sending these reports to the cancer registry center. In order to overcome these limitations, the cancer registry center of Mazandaran University of Medical Sciences performs population-based cancer registry, and in this way, many pathology reports of cancer patients are also received from non-pathology sources. Also, all received reports even if they are coded in the pathology centers, they are coded again in the cancer registry center.

In general, the quality of the coding of the pathological data of the cancer registry centers seems to be at an acceptable level. Therefore, reports resulting from coding can be cited and reliable for epidemiological studies related to cancer if their coding is done correctly and completely. In the reports in which topographical coding was done, the Kappa coefficient was higher compared to all the received reports. The most reports were on carcinoma morphology, and the Kappa coefficient in morphology codes has almost complete reliability. In terms of behavior coding, there was the most agreement on the category of benign cancers. The Kappa coefficient in given behavior codes have low reliability. The Kappa coefficient in behavior codes has low reliability. It is recommended to use specialized and trained forces and create motivation to perform more complete coding in order to increase the quality of coding of pathology reports and periodical inspections of pathology centers and provide feedback to them.

## Supporting information

S1 File (RAR)
